# Late progression of pediatric Kikuchi-Fujimoto disease to systemic lupus erythematosus: a case report and review of literature

**DOI:** 10.3389/fimmu.2025.1724306

**Published:** 2026-01-16

**Authors:** Salma Bessioud, Karima Tlili, Hager Barakizou, Sameh Mezri

**Affiliations:** 1 Hopital Militaire Principal d’Instruction de Tunis, Tunis, Tunisia; 2 Universite de Tunis El Manar Faculte de Medecine de Tunis, Tunis, Tunisia; 3 Hopital Militaire Principal d’Instruction de Tunis, Tunis,, Tunisia

**Keywords:** autoimmunity, case report, cervical lymphadenopathy, differential diagnosis, Kikuchi-Fujimoto disease, pediatrics, systemic lupus erythematosus

## Abstract

**Background:**

Kikuchi-Fujimoto disease (KFD) is a rare, self-limiting lymphadenitis predominantly affecting children and young adults, often mimicking lymphoma or tuberculous lymphadenitis. Although it usually resolves spontaneously, KFD has occasionally been associated with autoimmune disorders, particularly systemic lupus erythematosus (SLE).

**Case:**

We report a six-year-old girl presenting with persistent cervical lymphadenopathy, low-grade fever, fatigue, and weight loss. Laboratory evaluation showed mild leukopenia and elevated erythrocyte sedimentation rate, while autoimmune markers were initially negative. Excisional lymph node biopsy confirmed KFD. She responded well to initial oral corticosteroid therapy, with complete regression of lymphadenopathy, though two recurrences occurred during follow-up, managed by a short course of corticosteroids once and resolved spontaneously on the other occasion. Remarkably, 12 years later, she presented with fatigue, arthralgia, and malar rash. Laboratory evaluation revealed cytopenias, proteinuria, and positive ANA and anti-dsDNA antibodies, leading to a diagnosis of SLE.

**Conclusion:**

This case highlights the potential long-term autoimmune risk following KFD and emphasizes the importance of sustained clinical and immunological follow-up in pediatric patients for early detection and timely management.

## Highlights

- Long-Term Follow-Up: KFD can precede SLE even after a decade; sustained clinical and immunological monitoring is essential.
- High-Risk Features: Female sex, personal/family autoimmunity, recurrent lymphadenopathy, prolonged fever, or lab abnormalities (ANA positivity, low complement, cytopenias) may indicate higher risk of SLE.
- Early Intervention: Prompt recognition of new clinical or serological signs allows timely management, potentially preventing complications from evolving SLE.

## Background

Kikuchi-Fujimoto disease (KFD), also called necrotizing histiocytic lymphadenitis, is generally benign, albeit sometimes life-altering, primarily affecting young Asian women and rarely seen in children. It usually presents with cervical lymphadenopathy accompanied by vague symptoms such as arthralgia, fever, and/or fatigue. Laboratory tests are non-specific, making it an important alternative diagnosis to malignancies, notably lymphoma, and other causes of chronic lymphadenopathy, especially in pediatric patients (1).

Since its first description in 1972 (2), many cases have been reported showing a connection to or progression into autoimmune diseases, particularly systemic lupus erythematosus. The pathophysiological link remains unclear and debated. Proposed mechanisms include an underlying autoimmune predisposition (3, 4) or genetic susceptibility via an aberrant immune response to infectious agents.

In children, KFD is even more uncommon, often presenting with misleading features and a benign yet perplexing course. Several pediatric cases have shown severe manifestations, including macrophage activation syndrome, suggesting significant immune dysregulation (5, 6). Histological examination of lymph nodes, the only definitive diagnostic tool, reveals architectural disruption with karyorrhectic necrosis, infiltration by CD68+ histiocytes, and absence of neutrophils, distinguishing KFD from lymphoma or tuberculosis.

Recent literature increasingly highlights the potential association between KFD and SLE. Approximately 13% of patients reportedly develop secondary SLE (4). This association is supported by observations, including cases in which lupus follows shortly after KFD (7). Furthermore, some authors have reported overlap syndromes involving lupus and Sjögren’s syndrome following KFD, reinforcing the concept of a shared immunological background (8).

This case report highlights the possible association between KFD and SLE, even after a long period, and emphasizes the importance of prolonged follow-up. The work has been reported in line with the SCARE criteria.

## Case presentation

A six-year-old girl with no significant past medical history was referred for cervical lymphadenopathy persisting for several weeks, associated with low-grade intermittent fever, fatigue, and weight loss. On examination, firm, tender, and mobile posterior cervical lymph nodes measuring 2–4 cm were noted, without overlying skin changes or signs of ENT or systemic infection. Cervical ultrasonography revealed oval, hypoechoic lymph nodes with heterogeneous internal echotexture and poorly visualized hila, while color Doppler imaging showed reduced or peripheral vascularization without evidence of abscess or suppuration.

Given the clinical picture, hematologic malignancy was initially suspected. Laboratory investigations were performed (**Table 1**), and serologies for cytomegalovirus, Epstein-Barr virus, toxoplasmosis, and tuberculosis were negative. Chest radiography was unremarkable.

**Table 1:** Comparative laboratory data of initial KFD diagnosis vs during follow-up.

Parameter	Reference range	Initial diagnosis (KFD)	During follow-up (suspected SLE)	Comment on evolution
ESR	<20 mm/h	28 mm/h	64 mm/h	Significant increase indicating inflammation
CRP	<5 mg/L	2 mg/L	9 mg/L	Mild elevation during SLE onset
Hemoglobin	12–16 g/dL	12 g/dL	9.2 g/dL	Development of normocytic anemia
WBC	4.0–10.0 ×10^9^/L	3.2 ×10^9^/L	3.1 ×10^9^/L	Persistent mild leukopenia
Platelet Count	150–400 ×10^9^/L	150 ×10^9^/L	105 ×10^9^/L	Thrombocytopenia developed during SLE
Proteinuria (24 h)	<150 mg/24 h	–	1.2 g/24 h	New onset proteinuria suggesting renal involvement
Hematuria (microscopic)	Absent	–	Present	Indicates renal involvement
ANA	<1:80	–	1:640 (speckled)	Strongly positive, supports autoimmune activity
Anti-dsDNA	Negative	–	Positive	Confirms SLE seroconversion
Complement C3	0.9–1.8 g/L	–	0.7 g/L	Decreased complement levels in SLE
Complement C4	0.1–0.4 g/L	–	0.06 g/L	Decreased complement levels in SLE
Direct Coombs Test	Negative	–	Positive	Hemolysis present at SLE onset

Laboratory parameters at the time of initial Kikuchi-Fujimoto disease diagnosis and during follow-up at progression to systemic lupus erythematosus (SLE).

**Figure 1:**
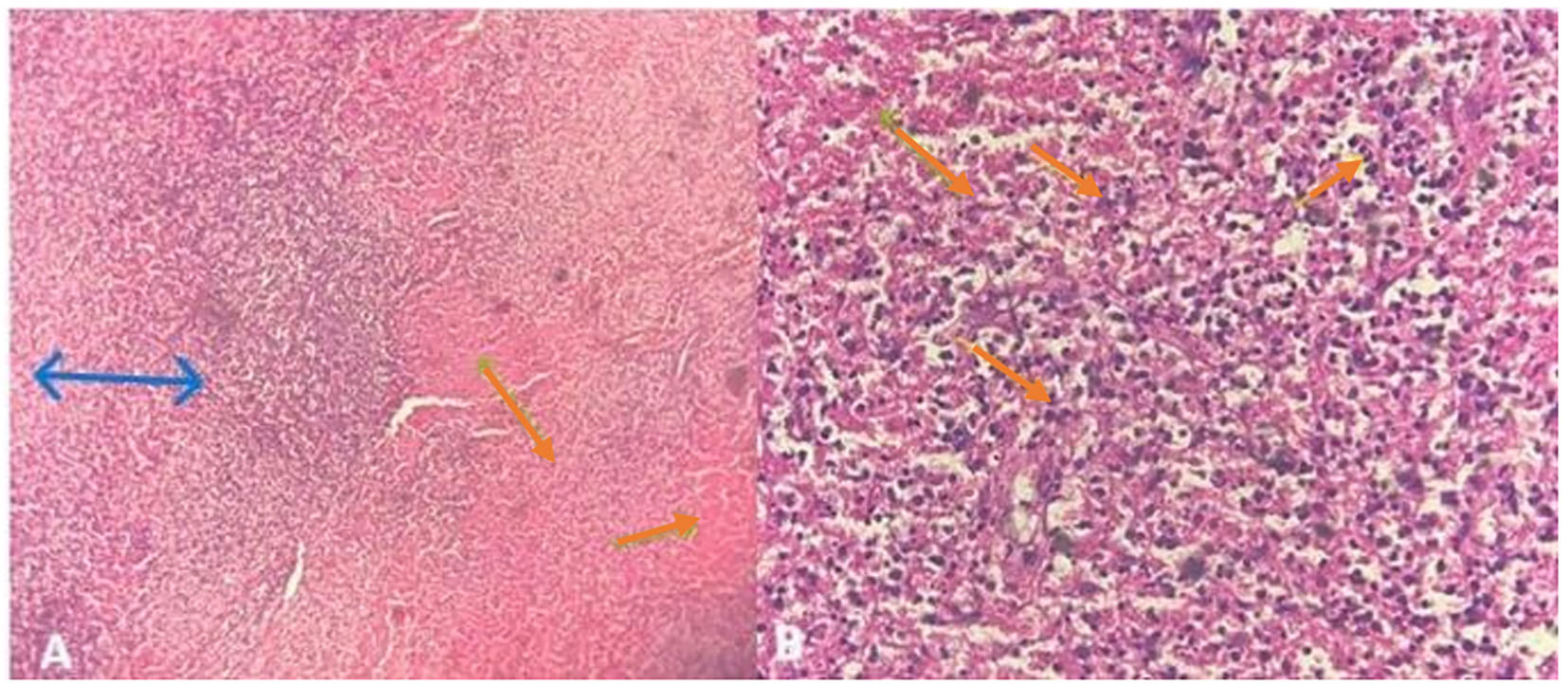
Histopathology of the excised cervical lymph node in Kikuchi-Fujimoto disease: **(A)** HEX4): Low-power view showing architectural distortion of the lymph node with large serpiginous-shaped pale areas. **(B)** HEX20): High-power view demonstrating pale areas composed of mononuclear cells, amorphous necrotic debris, and abundant karyorrhectic nuclear fragments, characteristic of necrotizing histiocytic lymphadenitis.

**Figure 2:**
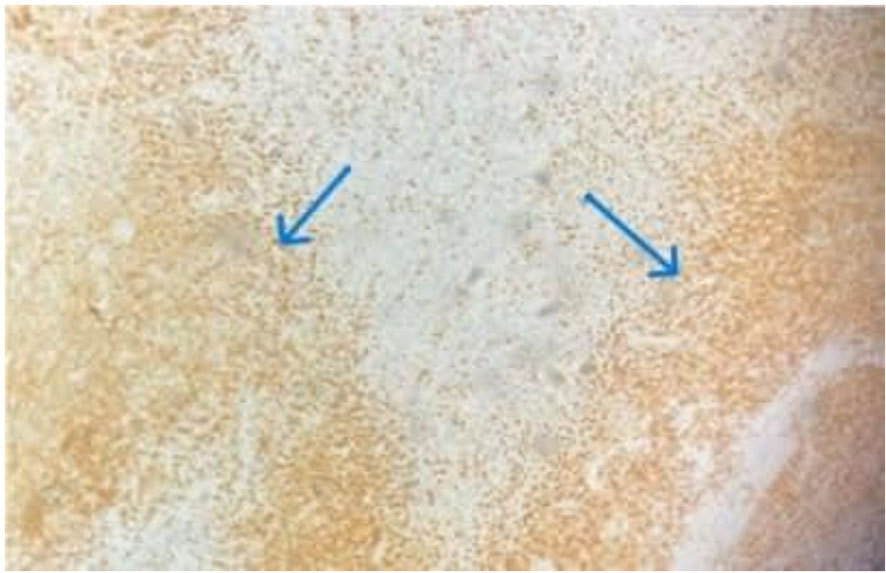
CD68 highlights abundant histiocytes surrounding areas of necrosis.

Excisional lymph node biopsy demonstrated disrupted architecture with extensive karyorrhectic necrosis, CD68+ histiocytic infiltration, plasmacytoid monocytes, numerous apoptotic cells, and an immunoblastic reaction, with absence of neutrophils or eosinophils, consistent with Kikuchi-Fujimoto disease (**Figures 1**, **2**).

The patient was treated with oral prednisone at 1 mg/kg/day for six weeks, followed by a gradual taper over two months, achieving complete regression of lymphadenopathy. During several years of clinical follow-up, two recurrences responded well to a short course of corticosteroids once and resolved spontaneously on the other occasion.

At 18 years of age, the patient presented with fatigue, arthralgia, and a persistent malar rash. Laboratory evaluation revealed evolving hematologic and immunologic abnormalities, including cytopenias, proteinuria, hematuria, high-titer speckled antinuclear antibodies, positive anti-dsDNA antibodies, decreased complement levels, and a positive direct Coombs test (**Table 1**), indicating transformation towards systemic lupus erythematosus (SLE).

**Figure 3:**
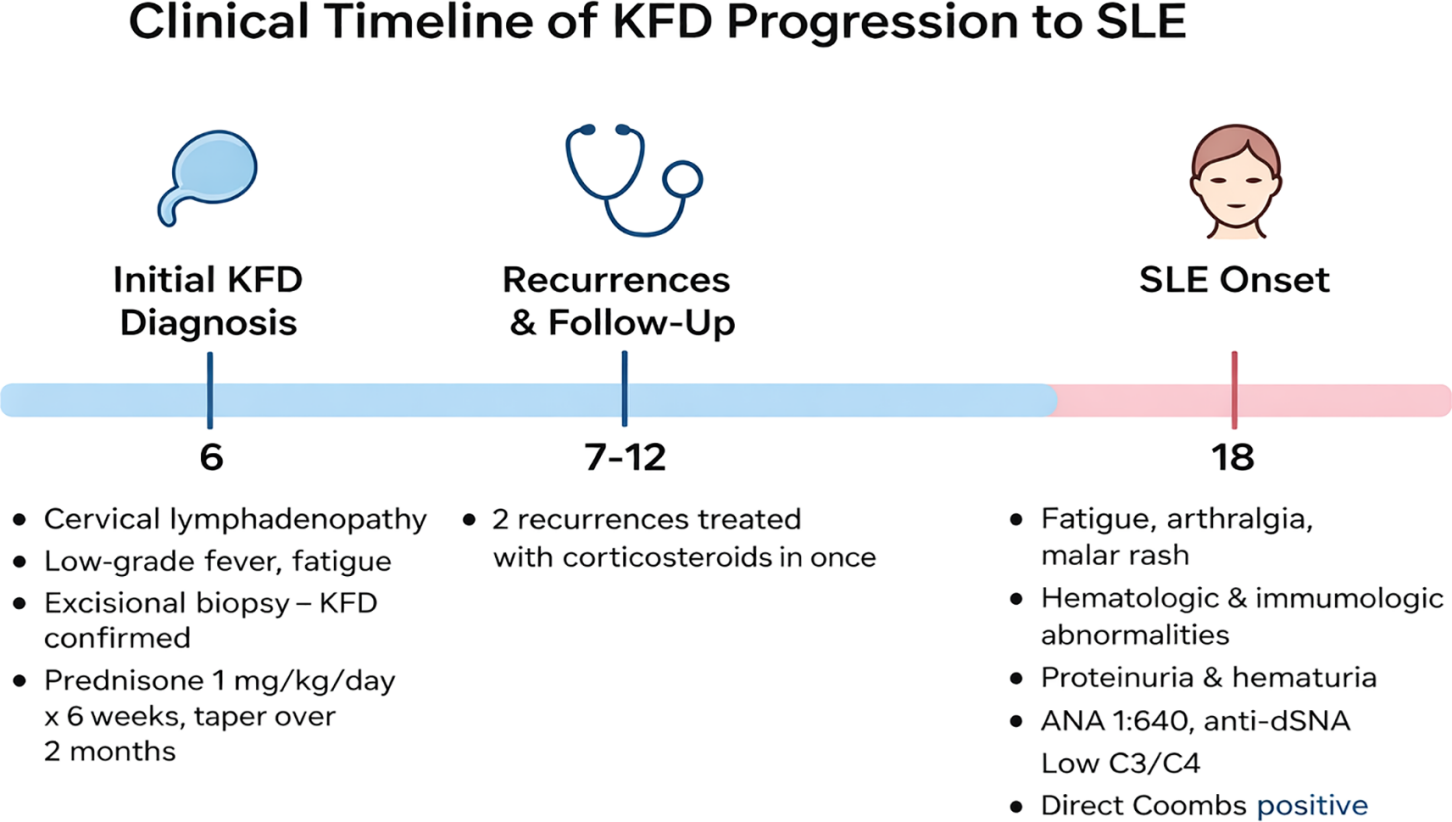
Timeline of diagnosis, relapses, follow-up, and onset of SLE.

According to the EULAR/ACR criteria, the diagnosis of systemic lupus erythematosus was established. Liver function tests, cardiac evaluation (echocardiogram), thoracic evaluation (chest X-ray), and electrolyte levels were normal, and no significant visceral involvement was noted at this stage (**Figure 3**).

The patient was referred to the internal medicine department. Treatment included 200 mg daily of hydroxychloroquine and 0.5 mg/kg/day of oral corticosteroids (prednisone*) due to hematologic and immunologic activity. Because of minimal visceral involvement, no additional immunosuppressive therapy was prescribed. Regular clinical and laboratory monitoring was implemented.

## Discussion

Kikuchi-Fujimoto disease (KFD) is a rare form of necrotizing histiocytic lymphadenitis. Its etiology remains unclear. Predominantly affecting young Asian females, recent pediatric cases have expanded the demographic spectrum (9, 10).

KFD usually presents with firm, tender, unilateral cervical lymphadenopathy, often associated with low-grade fever, asthenia, anorexia, and arthralgia. Due to its nonspecific clinical features, it is frequently misdiagnosed. Important differentials include lymphoma, lupus lymphadenitis, viral lymphadenitis, toxoplasmosis, and cat-scratch disease. Rosai–Dorfman disease (RDD) is particularly relevant because it closely mimics KFD; however, RDD typically manifests with massive, painless lymphadenopathy and demonstrates sinus histiocytosis with emperipolesis, along with S100 and CD68 positivity on immunohistochemistry (11, 12). Careful histopathological assessment is therefore essential to distinguish KFD from RDD and other mimickers.

Leukopenia was observed in our case and it is indeed well-described in KFD, occurring in approximately 20–58% of cases It is believed to result from immune-mediated cytotoxicity and increased apoptosis of lymphoid cells (11).

Histopathology remains crucial for KFD diagnosis, showing disrupted lymph node architecture with patchy karyorrhectic necrosis, CD68+ histiocytes, plasmacytoid monocytes, absence of neutrophils and eosinophils, and a predominance of CD8 over CD4 T-cell infiltrates. Immunohistochemistry can help differentiate KFD from lymphoma and RDD, and excisional biopsy is preferred for diagnostic accuracy (13, 14). These features highlight the importance of considering both clinical presentation and histopathological findings when evaluating suspected cases of KFD.

In children, KFD is rare and represents a considerable diagnostic challenge. Wang et al. (12) reported that its misleading presentation frequently necessitated invasive procedures to exclude lymphoma, while more recent pediatric cases described by Liu et al. (5) and Shen et al. (6) have shown complications such as macrophage activation syndrome, suggesting an exaggerated immune response possibly linked to autoimmune dysregulation. (**Table 2**).

**Table 2:** Comparative table of typical pediatric KFD versus index case.

Parameter	Typical pediatric KFD	Index case
Age	10–25 years	12 years
Sex	Female > Male	Female
Symptoms	Fever, cervical lymphadenopathy, fatigue, arthralgia	Fever, tender cervical lymphadenopathy, asthenia
Laboratory Findings	Mild leukopenia, elevated ESR, ANA usually negative	Mild leukopenia, elevated ESR, ANA initially negative
Histopathology	Karyorrhectic necrosis, CD68+ histiocytes, plasmacytoid monocytes, absent neutrophils	Same pattern confirmed by lymph node biopsy
Treatment	Supportive care; corticosteroids in severe cases	Supportive care; corticosteroids
Outcome	Usually self-limiting	Complete resolution of KFD; later developed SLE after 12 years

KFD is benign and self-limiting (12, 13). It does not confer immunodeficiency, and opportunistic infections are very rare, primarily when patients receive immunosuppressive therapy such as corticosteroids or hydroxychloroquine for severe KFD or a subsequent autoimmune condition (12). Likewise, there is no documented risk of malignant transformation, although careful evaluation including lymph node biopsy and immunohistochemistry is essential to exclude lymphoma at initial presentation (12, 13).

Yousefi et al. (7) described a case of near-simultaneous onset in an adolescent, while Cindy et al. (8) reported an initial KFD presentation in a young girl who developed SLE in one year. In our case, the patient was a six-year-old girl, consistent with the reported female predominance in pediatric KFD.

While specific genetic testing was not conducted in this case, literature suggests a potential genetic predisposition to autoimmune diseases in KFD patients. Abnormalities in the complement system and the presence of antinuclear antibodies may contribute to progression from KFD to SLE (14). More recent investigations using advanced genetic testing have highlighted potential associations with HLA class II variants and other susceptibility loci in patients with KFD who later develop SLE, suggesting a heritable immunogenetic predisposition (11, 15).

In our case, the 12-year interval between KFD diagnosis and SLE onset emphasizes the potentially latent nature of this progression, longer than in most previously reported pediatric cases (**Table 3**). Deb et al. (11) noted that SLE onset could be delayed by several years and stressed the importance of long-term follow-up even in cases with complete clinical remission. Similarly, Owczarczyk-Saczonek et al. (16) reported the emergence of chronic cutaneous lupus following isolated KFD, suggesting a possible underlying immunological continuum.

**Table 3:** Comparison of typical pediatric KFD features vs index case.

Study	Age/Sex	Interval to SLE	Clinical features	Outcome
Yoon et al. (2004) (17)	14/F	2 years	Recurrent fever, lymphadenopathy, photosensitivity	Developed SLE with renal involvement
Baenas et al. (2016) (14)	27/F	3 years	Arthralgia, malar rash, positive ANA	Diagnosed with SLE; treated with immunosuppressants
Qin et al. (2024) (18)	18/F	5 years	Neurological symptoms, venous sinus thrombosis	Diagnosed with neuropsychiatric SLE; treated with corticosteroids
Decker et al. (2023) (19)	16/F	6 years	Acneiform eruption, positive ANA	Diagnosed with SLE; managed with hydroxychloroquine
Our case (Index case)	12/F	12 years	Skin symptoms, biological data indicating SLE	Diagnosed with SLE; treated with corticosteroids

Serial ANA, anti-dsDNA, and complement measurements were not performed routinely because the patient remained clinically asymptomatic for more than a decade after KFD resolution,. At SLE onset, however, she exhibited sudden seroconversion with high-titer ANA, positive anti-dsDNA, and hypocomplementemia. This pattern suggests an abrupt immunological transition rather than a slow, detectable progression. Longitudinal serologic monitoring in future cohorts may help identify early predictors of autoimmune evolution.

These observations highlight the differences and similarities between our index case and previously reported pediatric cases, particularly regarding the interval to SLE onset, initial presentation, and laboratory features. The unusually long latency underscores the need for sustained follow-up. (**Table 3**).

The pathogenesis of KFD involves aberrant T-cell activation, particularly CD8+ cytotoxic lymphocytes, which mediate histiocytic necrosis in affected lymph nodes (12, 13). Cytokines such as interferon-gamma (IFN-γ) and interleukin-6 (IL-6) have been implicated in this immune-mediated process (12). In genetically predisposed individuals, including those with complement pathway abnormalities or transient antinuclear autoantibodies, KFD may represent an early, subclinical phase of immune dysregulation, evolving into SLE over time.

Currently, no validated prediction score exists to identify which KFD patients will progress to SLE. Observational data suggest that female sex, personal or family history of autoimmunity, recurrent lymphadenopathy, prolonged fever, arthralgia, or laboratory abnormalities (ANA positivity, low complement, cytopenias) may indicate higher risk. Histopathological features, such as prominent plasmacytoid dendritic cell infiltrates, may further reflect underlying autoimmune activation (4, 16). Long-term clinical and serological follow-up is recommended to allow early recognition and timely management.

There are no standardized recommendations for KFD follow-up in pediatric patients. However, many studies underline the value of long-term clinical and immunological monitoring, particularly in females due to the frequency of emergence of autoimmune conditions like lupus. Clinicians should remain vigilant for unexplained fatigue, arthralgia, or skin manifestations, and assess ANA levels, complement activity, and hematological parameters.

Management becomes more complex when a patient later develops SLE. In our case, hydroxychloroquine combined with corticosteroids led to noticeable improvement, echoing other early lupus cases that followed KFD (7, 8).

Compared with previously reported pediatric cases, this patient demonstrates an unusually long interval between KFD and subsequent SLE, emphasizing the variable latency of autoimmune evolution and underscoring the need to consider long-term follow-up even in apparently resolved cases.

### Conclusion

Although generally self-limiting, Kikuchi-Fujimoto disease (KFD) warrants long-term clinical and immunological follow-up in children due to its potential progression to systemic lupus erythematosus (SLE). Careful monitoring allows early detection of autoimmune evolution and timely management.

## Data Availability

The raw data supporting the conclusions of this article will be made available by the authors, without undue reservation.
